# Decomposition of caste differential in life satisfaction among older adults in India

**DOI:** 10.1186/s12877-022-03526-1

**Published:** 2022-11-02

**Authors:** T. Muhammad, Ronak Paul, Trupti Meher, Rashmi Rashmi, Shobhit Srivastava

**Affiliations:** International Institute for Population Sciences, Mumbai, -400088 India

**Keywords:** Caste, Life satisfaction, Older adults, India

## Abstract

**Background:**

Being a multi-cultured country, India has varied social groups which largely shape the lives of individuals. Literature has shown that life satisfaction is highly associated with the social status of individuals. However, changing age dynamics (growing older) and definition of life among people presses the need to understand whether the additional years of life in older adults are manifested with the disparity in life satisfaction among the Scheduled Caste (SC)/ Scheduled Tribes (ST) and non-SC/ST social groups in recent years. The present study explored the factors contributing to such differences in life satisfaction across social groups.

**Methods:**

This study used data from the Longitudinal Aging Study in India conducted during 2017-18. The analytical sample of the study was 30,370 older adults. Life satisfaction was the outcome variable with a score ranging from 5 to 35. Descriptive statistics and bivariate analysis were conducted. Simple linear regression analysis was used to establish the association between the outcome and explanatory variables. Further, the Blinder-Oaxaca decomposition model was used to analyse the role of explanatory factors in the caste difference in life satisfaction among older people.

**Results:**

Overall, the life satisfaction score among older adults in the study was 23.9 (SD- 7.3). Older adults from non-SC/ST group had significantly higher likelihood of having life satisfaction in comparison to older adults from SC/ST group [Coef: 0.31; CI: 0.14, 0.49]. The decomposition results showed that the model explained 74.3% of the caste gap (between SC/ST and non-SC/ST) in life satisfaction among older adults in India. Subjective social status (39.0%) was widening the gap for life satisfaction among older adults from SC/ST and non-SC/ST group. Similarly, level of education (15.2%) followed by satisfaction with living arrangement (13.2%) and place of residence (5.3%) contributed for widening the gap for life satisfaction among older adults from SC/ST and non-SC/ST group. Region of country (− 11.5%) followed by self-rated health (− 3.0%) and major depression (− 2.7%) contributed for narrowing down the gap for life satisfaction among older adults from SC/ST and non-SC/ST group.

**Conclusion:**

Older adults belonging to non-SC/ST groups were more likely to have a higher level of life satisfaction than those from the SC/ST group. Factors like subjective social status, educational level, living arrangement satisfaction, and place of residence explained the caste differential in life satisfaction among older adults. In addition, factors such as psychological health and perceived health status should be the area of concern and special focus for policy makers and researchers in terms of reducing social inequalities in wellbeing among older population.

## Background

Individuals’ deteriorated subjective wellbeing has often been a concern on their healthier longevity [1]. One of the greatest contributors to subjective wellbeing, life satisfaction, is an individual’s judgment of wellbeing and quality of life [2, 3]. Though the major shift in life role occurs at old age in the form of health, work status, household headship, autonomy accompanied by psychological distress [4–6]. Life satisfaction is highly associated with old-age [3]. Although individual’s life satisfaction can be associated with their interpretation of present or past life and their criteria for satisfaction and happiness [7]. Research has also linked social class with life satisfaction [8, 9]. Evidence shows that upper-class individuals are more satisfied in their lives than bottom-class individuals as they have better education, healthier and longer life, economic wellness, and more opportunities for their children [10–13]. Intergenerational downward mobility in education and income was highly associated with life satisfaction [14, 15]. However, ample evidence found that those in higher working classes often suffer from dissatisfaction in their lives [16–19].

Being a multi-cultured country, India has varied social groups, namely Other Backward Class (OBC), General/ Others, Scheduled caste (SC), and Scheduled tribes (ST), divided as per social stratification [20]. With 16.6 and 8.6% of the total population, SC and ST are considered disadvantaged socio-economic groups, respectively [21]. Different measures have tried to bring equity in the living condition and health of lower-class individuals; however, constant discrimination and deprivation blur such effort. Besides caste being a prominent indicator of wellbeing in India, a question arises whether caste also affects the individual’s life satisfaction. Is there any disparity in evaluating life through “the thoughts that people have about their life when they think about it” across the SC/ST and OBC/other groups? If yes, then who is more satisfied; those with higher privileges (non-SC/ST) or those who face discrimination (SC/ST).

Notably, the meaning of life satisfaction in older people is entirely different from those in the working or younger age group. Old age life satisfaction is often associated with living arrangement, social participation, and the care they receive from their family members [22, 23]. However, such factors may vary across older adults belonging SC/ST and non-SC/ST groups in India, leading to cross-cultural variations in life satisfaction [24]. For instance, SC/ST populations are mostly restricted to nature’s resources for their daily requirement [25]. The older adults in SC/ST groups are economically disadvantaged with lesser opportunities, but the strong community network can help in their upliftment [26–28]. On the other hand, the non-SC/ST population who enjoy a prosperous life are usually not required to do all the difficult chores [27]. However, their children often leave them behind, leading to social isolation, and the changing living arrangements which are often associated with subjective wellbeing [29, 30].

**Fig. 1:**
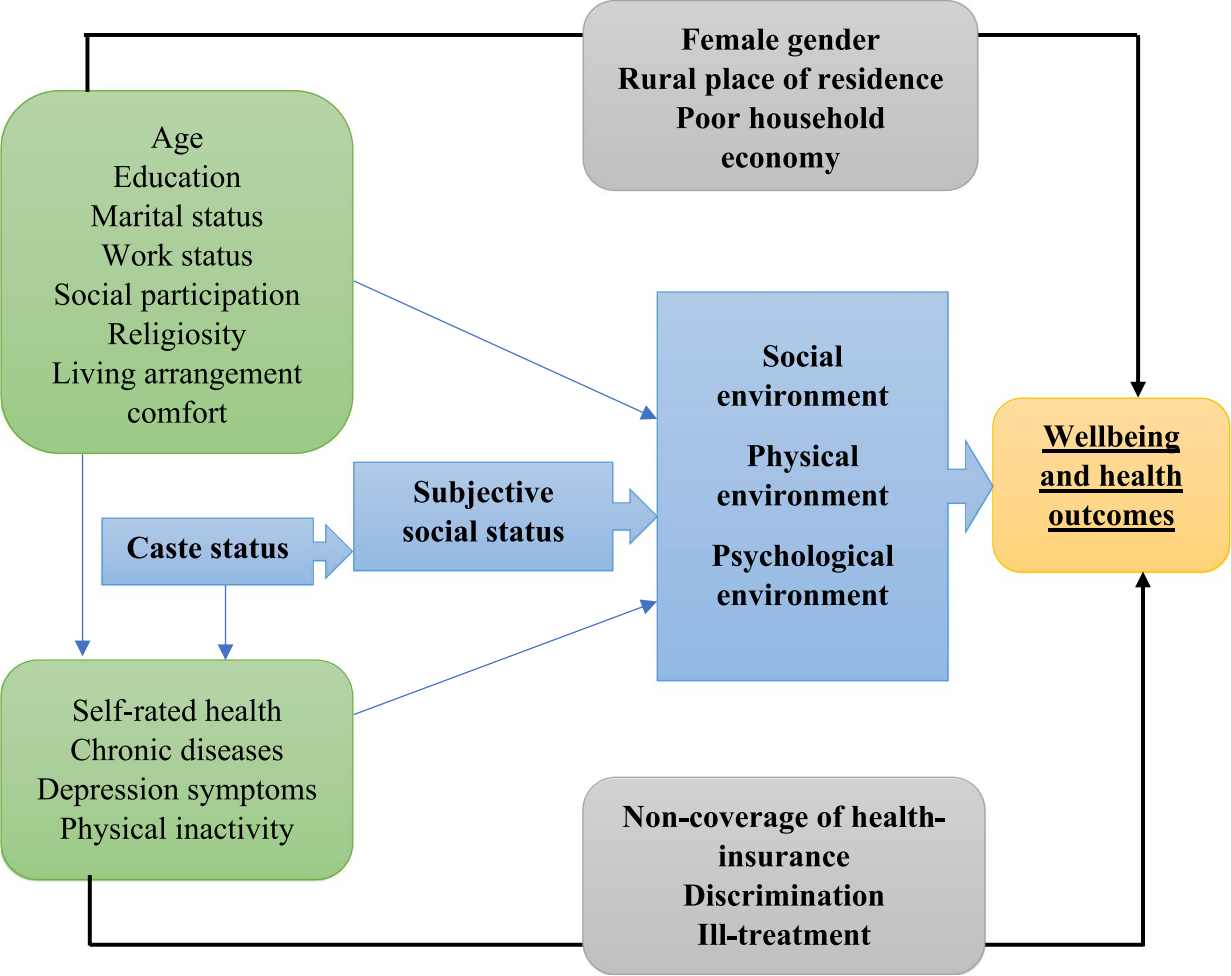
Theoretical framework

Although literature shows lower life satisfaction among under-privileged social groups in India due to their long-standing history of deprivation and discrimination [31, 32]. A recent evidence found that the average life satisfaction of individuals belonging to OBC was lowest compared to those from SC/ST individuals [33]. The changing definition of happiness and life evaluation among SC/ST and non-SC/ST populations in India presses the need to understand whether the additional years of life in older adults are manifested with the disparity in life satisfaction among the social groups in recent years. Topics like subjective well-being, mental health, life satisfaction have gained wide recognition in India [34, 35]. However, their better measurement and associated factors remained poorly understood. The wide source of information about participants’ socio-demographic, health and household characteristics from the recently released data of the Longitudinal Aging Study in India (LASI) brings an advantage to overcome those issues. Thus, the present study adds prominent evidence by exploring how life satisfaction varies across SC/ST and non-SC/ST population sub-groups. We hypothesize that there is a significant difference in life satisfaction of SC/ST and non-SC/ST older adults in India. Besides understanding the disparities across the social groups, present study will be helpful for the policymakers to identify the factors leading to such differences. Thus, a decomposition technique is used to investigate the factors contributing to differences in life satisfaction across social groups. Based on the findings from existing studies, a theoretical framework has been developed and summarised in Fig. 1.

## Methods

### Data

This study used data from LASI, wave-I conducted during 2017-18. The LASI, which is the Indian version of the Health and Retirement Studies (HRS), is a nationally representative survey conducted by the International Institute for Population Sciences (IIPS) in collaboration with the Harvard T.H. Chan School of Public Health, and the University of Southern California (USC). LASI provides vital information on demography, chronic health conditions, symptom-based health conditions, functional health, mental health (cognition and depression), household economic status, healthcare utilization and health insurance, family and social networks, work and employment, retirement and life expectations of 72,250 adults aged 45 and above across all the states and union territories of India. LASI adopted a multistage stratified cluster sampling design intending to follow the sample biennially for 25 years. Further details regarding the sample design, survey instruments, fieldwork, data collection and processing, and response rates are publicly available elsewhere [36]. All methods were performed in accordance with the relevant guidelines and regulations.

The current study is based on a sample of 31,464 older adults in India who were aged 60 years and above. However, information on life satisfaction score was unavailable for 1094 older adults. Therefore, the analytical sample of the current study is 30,370 older adults among whom 9935 and 20,435 belonged to the SC-ST group and non-SC-ST group respectively.

### Outcome variable

The continuous measure of life satisfaction score is the outcome variable of this study. During LASI wave-I, the interviewers collected information on the following five life satisfaction indicators:

- In most ways, the respondents’ life is close to ideal.
- The conditions of the respondents’ life are excellent.
- The respondents are satisfied with their life.
- The respondents have achieved the essential things they want in their life so far.
- If the respondents could relive their life, they would change almost nothing.

The responses to these questions were recorded as strongly disagree (scored as 1), somewhat disagree (score 2), slightly disagree (score 3), neither agree nor disagree (score 4), slightly agree (score 5), somewhat agree (score 6) and strongly agree (score 7). Notably, all the five life satisfaction indicators had an excellent Cronbach’s alpha value of 0.90. Therefore, we summed the five indicators to obtain the life satisfaction score of older adults ranging from 5 to 35.

### Group variable

The caste group was coded as Scheduled Caste/Scheduled Tribe (SC/ST) and non-SC/ST.

### Explanatory variables

Based on existing research, we included the following socio-demographic, health-related and household characteristics in this study [22, 31, 33]. The socio-demographic characteristics of older adults include: age denoted as young-old (aged 60-69 years), old-old (aged 70-79 years) and oldest-old (aged 80 years or more); gender (male/female); marital status (currently married/currently not married); level of education (no formal education, up to primary, secondary and above); working status (never worked, currently not working, currently working, retired); subjective social status (very low, low, medium, high and very high); social participation (socially active/socially inactive); importance of religion (not important/very important); living arrangement satisfaction (satisfied, neutral, not satisfied); faces discrimination in life (no/yes); received ill-treatment within 1 year from the interview (no/yes).

Next, the health-related characteristics of the older adults were – self-rated health (good, average, poor); chronic morbidity status (no condition, single condition and multiple conditions) based on ever-diagnosed disease information in LASI; depression (not diagnosed /diagnosed); physical activity status (physically inactive/physically active); difficulty in Activities of Daily Living (ADL) (no/yes); difficulty in Instrumental Activities of Daily Living (IADL) (no/yes); covered by any health insurance (yes/no).

Further, the household-related characteristics were – monthly per capita consumpsion expenditure (MPCE) quintile of household (poorest, poorer, middle, richer and richest); religion (Hinduism, Islam, Others); place of residence (urban/rural); regions (Southern, Northern, Central, Western, Eastern, North-eastern).

### Statistical methods

We started by showing the absolute and weighted percentage distributions of older people depending on their background variables. The proportion test was used to look at the difference in life satisfaction between older adults from SC/ST and non-SC/ST groups. Simple linear regression analysis was used to establish the association between the outcome and explanatory variables. The coefficients along with 95% confidence interval (CI) were presented.

We used the Blinder-Oaxaca decomposition approach to investigate the importance of factors explaining life satisfaction inequality between the SC/ST and non-SC/ST groups.

The Blinder-Oaxaca decomposition approach is important in this study because it can distinguish outcomes attributed to unequal treatment across groups from outcomes attributable to group differences in fundamental traits like role and experience. This technique allowed us to distinguish between two causes of the outcome disparity between SC/ST and non-SC/ST older adults: (i) variations in the distribution of factors between the groups, and (ii) differences in the impact of these variables between the groups.

Following the decomposition analysis, two separate equations of the semi-log functional form for life satisfaction for SC/ST and non-SC/ST older adults are estimated based on ordinary least squares (OLS):

$${e}_{si}=\ln \left({y}_{si}\right)={\beta}_s{X}_{si}+{\varepsilon}_i,$$

$${e}_{ni}=\ln \left({y}_{ni}\right)={\beta}_n{X}_{ni}+{\varepsilon}_i,$$

Where y_ji_ is the life satisfaction score for j^th^ type (j = (s, n), s denoting SC/ST and n denoting non-SC/ST) of i^th^ member, X_i_ is a vector consisting of background characteristics corresponding to the member i.

The average gap in life satisfaction score can be divided into explained and unexplained components as:

$${\overline{e}}_s-{\overline{e}}_n=\left({\overline{X}}_s-{\overline{X}}_n\right){\hat{\beta}}_s+{\overline{X}}_n\left({\hat{\beta}}_s-{\hat{\beta}}_n\right)=E+D$$

Where, the first component (E) measures the inequality due to the differences between SC/ST and non-SC/ST older adults in observed characteristics (characteristics effect). In other words, the expected difference in life satisfaction score if the non-SC/ST older adults were given SC/ST older adults characteristics. The second component (D) measures the inequality due to the different effects of the observed characteristics on SC/ST and non-SC/ST older adults (coefficients effect).

There was no evidence of multicollinearity between the independent variables when the variance inflation factor was calculated. The studies presented in the following sections were conducted using STATA 14. Because the LASI used a multi-stage sampling technique, standard errors for weighting and clustering were adjusted in all estimations.

## Results

**Fig. 2:**
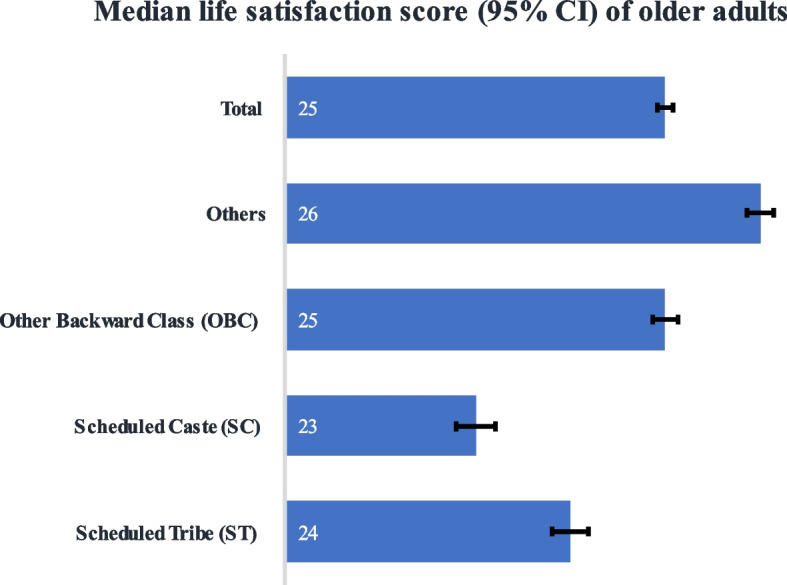
Median life satisfaction score of older adults (60+ years) by caste groups in India

Figure 2 represents the median life satisfaction score of older adults (60+ years) by caste groups in India. The median life satisfaction score was 25 among older adults in India. The median score was lowest among older adults from Scheduled Caste (23) followed by Scheduled Tribe (24).

**Fig. 3:**
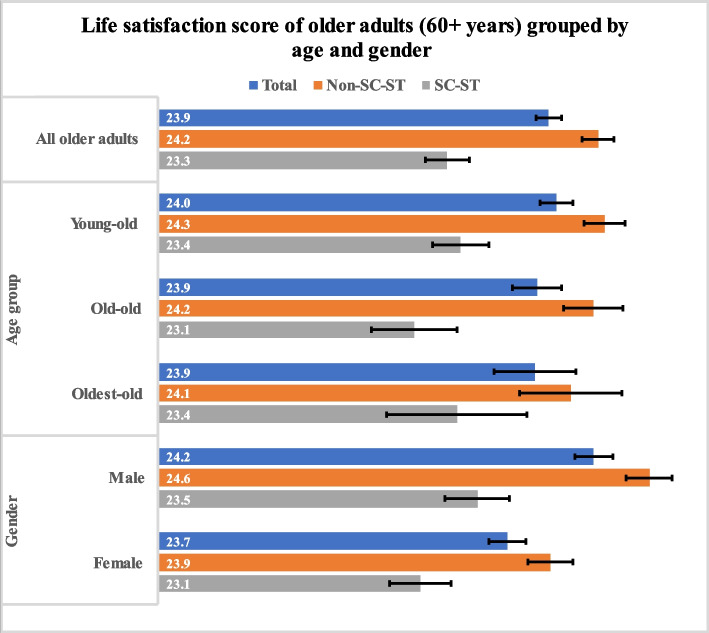
Life satisfaction score of all, young-old, old-old, oldest-old, male and female older adults across caste groups in India 2017-18

Figure 3 represents the life satisfaction score of all, young-old, old-old, oldest-old, male and female older adults across caste groups in India. It was found that the mean life satisfaction score was low among older adults from SC-ST caste group (23.3) in reference to older adults from non-SC-ST caste group (24.2). The similar trend was observed in age group and gender.

Table 1 represents the socio-demographic, health-related, and household characteristics profile of older adults in India. About 27.1% of older adults belonged to SC-ST category. Overall, life satisfaction score among older adults in the study was 23.9 (SD- 7.3). Among the current sample of older adults, 38.2% were not currently married (SC/ST: 39.4% and non-SC/ST: 37.7%], and 56.4% older adults had no formal education (SC/ST: 72.4% and non-SC/ST: 50.5%]. A proportion of 30.5% older adults were currently working (SC/ST: 37.8% and non-SC/ST: 27.8%]. About 21.0% older adults had very low subjective social status (SC/ST: 30.6% and non-SC/ST: 17.4%]. Nearly 8.7% older adults were socially inactive (SC/ST: 10.0% and non-SC/ST: 8.2%]. Also, 21.3% older adults reported that religion was not important to them (SC/ST: 26.8% and non-SC/ST: 19.2%]. A proportion of 5.6% older adults were not satisfied with their living arrangement (SC/ST: 7.6% and non-SC/ST: 4.9%].

**Table 1:** Socio-demographic, health-related, and household characteristics profile of older adults in India 2017-18

Characteristics	Older adults (60+ years)						
	All		SC/ST group		Non-SC/ST group		Caste difference
	Mean/N	SD/WC_%	Mean/N	SD/WC_%	Mean/N	SD/WC_%	***p***-value
**Life satisfaction score**	23.9	7.3	23.3	7.1	24.2	7.3	< 0.001
**Caste of household**							
SC/ST group	9935	27.1	–	–	–	–	
Non-SC/ST group	20,435	72.9	–	–	–	–	
***Socio-demographic characteristics***							
**Age group**							
Young-old	18,525	59.5	6221	61.8	12,304	58.6	< 0.001
Old-old	8753	29.8	2751	28.5	6002	30.3	
Oldest-old	3092	10.7	963	9.7	2129	11.1	
**Gender**							
Male	14,553	47.2	4715	46.7	9838	47.4	0.263
Female	15,817	52.8	5220	53.3	10,597	52.6	
**Current marital status**							
Currently not married	11,004	38.2	3758	39.4	7246	37.7	< 0.001
Currently married	19,366	61.8	6177	60.6	13,189	62.3	
**Level of education**							
No formal education	16,224	56.4	6572	72.4	9652	50.5	< 0.001
Upto primary	7330	22.8	2144	16.6	5186	25.1	
Secondary and above	6816	20.8	1219	10.9	5597	24.4	
**Working status**							
Currently working	8798	30.5	3535	37.8	5263	27.8	< 0.001
Currently not working	10,475	35.6	3473	37.7	7002	34.8	
Never worked	8489	26.5	2253	18.9	6236	29.4	
Retired	2608	7.3	674	5.6	1934	8.0	
**Subjective social status**							
Very low	5384	21.0	2245	30.6	3139	17.4	< 0.001
Low	11,137	36.7	4075	40.0	7062	35.5	
Medium	9267	28.3	2585	21.2	6682	30.9	
High	3678	10.9	834	6.5	2844	12.5	
Very high	904	3.2	196	1.7	708	3.7	
**Social participation**							
Socially active	27,957	91.3	9012	90.0	18,945	91.8	< 0.001
Socially inactive	2413	8.7	923	10.0	1490	8.2	
**Importance of religion**							
Not important	5959	21.3	2200	26.8	3759	19.2	< 0.001
Very important	24,411	78.7	7735	73.2	16,676	80.8	
**Living arrangement satisfaction**							
Not satisfied	1417	5.6	501	7.6	916	4.9	< 0.001
Neutral	5160	19.4	1883	22.0	3277	18.4	
Satisfied	23,793	75.0	7551	70.3	16,242	76.7	
**Faces discrimination in life**							
Yes	4662	17.6	1475	19.5	3187	16.9	0.089
No	25,708	82.4	8460	80.5	17,248	83.1	
**Received ill-treatment**							
Yes	1264	5.2	426	6.0	838	4.9	0.444
No	29,106	94.8	9509	94.0	19,597	95.1	
***Health characteristics***							
**Self-rated health**							
Poor	6663	23.1	1948	24.0	4715	22.7	< 0.001
Average	19,420	63.3	6578	63.7	12,842	63.1	
Good	4287	13.6	1409	12.3	2878	14.1	
**Chronic morbidity status**							
No condition	13,929	47.1	5537	55.2	8392	44.0	< 0.001
Single condition	8957	29.1	2702	28.0	6255	29.6	
Multiple conditions	7484	23.8	1696	16.8	5788	26.4	
**Major depression**							
Depressed	2101	8.5	582	8.4	1519	8.5	< 0.001
Not depressed	28,269	91.5	9353	91.6	18,916	91.5	
**Physical activity status**							
Physically active	7951	27.6	2909	30.6	5042	26.5	< 0.001
Physically inactive	22,419	72.4	7026	69.4	15,393	73.5	
**Difficulty in ADL**							
Faces difficulty	6207	22.7	1834	23.1	4373	22.5	< 0.001
No difficulty	24,163	77.3	8101	76.9	16,062	77.5	
**Difficulty in IADL**							
Faces difficulty	13,186	47.5	4205	47.9	8981	47.3	0.007
No difficulty	17,184	52.5	5730	52.1	11,454	52.7	
**Covered by health insurance**							
Yes	6424	18.4	2462	21.5	3962	17.2	< 0.001
No	23,946	81.6	7473	78.5	16,473	82.8	
***Household characteristics***							
**Household MPCE Quintile**							
Poorest	6221	21.8	2758	29.9	3463	18.7	< 0.001
Poorer	6248	21.8	2167	24.4	4081	20.8	
Middle	6203	20.7	1958	19.2	4245	21.3	
Richer	5974	19.2	1704	15.6	4270	20.6	
Richest	5724	16.5	1348	10.8	4376	18.6	
**Religion of household**							
Hinduism	22,283	82.7	6264	84.7	16,019	82.0	< 0.001
Islam	3582	10.7	635	1.5	2947	14.1	
Others	4505	6.6	3036	13.7	1469	3.9	
**Place of residence**							
Rural	20,036	71.1	7627	84.3	12,409	66.2	< 0.001
Urban	10,334	28.9	2308	15.7	8026	33.8	
**Country region**							
Southern	7291	22.2	1792	14.7	5499	24.9	< 0.001
Northern	7726	25.2	2184	27.3	5542	24.4	
Central	2017	8.5	641	10.1	1376	8.0	
Western	4133	17.2	1206	16.0	2927	17.7	
Eastern	5601	23.9	1674	27.4	3927	22.6	
North-eastern	3602	3.0	2438	4.6	1164	2.4	
**Overall**	**30,370**	**100.0**	**9935**	**100.0**	**20,435**	**100.0**	

Note – (a) *N* Sample size, *SD* Standard deviation, *WC_%* Weighted column percentage; (b) Caste difference of life satisfaction score was tested using Mann-Whitney-Wilcoxon test while the caste difference of explanatory variables was tested using the chi-square test for independence; (c) *ADL* Activities of daily living, *IADL* Instrumental activities of daily living, *MPCE* Monthly per capita consumption expenditure

Table 2 presents the linear regression model showing multivariate association between life satisfaction and socio-demographic, health-related and household characteristics of older adults in India. It was revealed that older adults from non-SC/ST group had significantly higher likelihood for having higher life satisfaction in comparison to older adults from SC/ST group [Coef: 0.31; CI: 0.14, 0.49].

**Table 2:** Linear regression models showing multivariate association between life satisfaction and socio-demographic, health-related and household characteristics of older adults in India 2017-18

Characteristics	Life satisfaction among older adults (60+ years)					
	All		SC-ST group		Non-SC-ST group	
	Coefficient	95% CI	Coefficient	95% CI	Coefficient	95% CI
**Caste of household**						
SC/ST group	(Ref)					
Non-SC/ST group	0.31***	(0.14, 0.49)	–	–	–	–
***Socio-demographic characteristics***						
**Age group**						
Young-old	(Ref)		(Ref)		(Ref)	
Old-old	0.19**	(0.02, 0.36)	0.12	(−0.17, 0.42)	0.21*	(0.00, 0.42)
Oldest-old	0.64***	(0.37, 0.91)	0.86***	(0.40, 1.32)	0.55***	(0.22, 0.87)
**Gender**						
Male	(Ref)		(Ref)		(Ref)	
Female	0.28***	(0.08, 0.47)	0.43***	(0.13, 0.74)	0.17	(− 0.08, 0.42)
**Current marital status**						
Currently not married	(Ref)		(Ref)		(Ref)	
Currently married	0.32***	(0.15, 0.49)	0.31**	(0.02, 0.60)	0.30***	(0.09, 0.51)
**Level of education**						
No formal education	(Ref)		(Ref)		(Ref)	
Upto primary	0.41***	(0.22, 0.60)	0.80***	(0.46, 1.14)	0.23*	(−0.01, 0.47)
Secondary and above	0.82***	(0.59, 1.05)	0.87***	(0.40, 1.33)	0.73***	(0.46, 1.01)
**Working status**						
Currently working	(Ref)		(Ref)		(Ref)	
Currently not working	−0.073	(− 0.28, 0.13)	− 0.40**	(− 0.74, − 0.07)	0.12	(−0.13, 0.38)
Never worked	−0.044	(−0.28, 0.19)	− 0.31	(− 0.70, 0.09)	0.14	(− 0.16, 0.44)
Retired	0.64***	(0.32, 0.95)	0.27	(−0.32, 0.85)	0.80***	(0.43, 1.17)
**Subjective social status**						
Very low	(Ref)		(Ref)		(Ref)	
Low	0.99***	(0.77, 1.20)	1.33***	(0.99, 1.67)	0.74***	(0.46, 1.02)
Medium	2.66***	(2.42, 2.89)	3.19***	(2.80, 3.57)	2.37***	(2.07, 2.67)
High	3.37***	(3.08, 3.67)	3.39***	(2.85, 3.93)	3.25***	(2.89, 3.61)
Very high	5.00***	(4.52, 5.47)	3.77***	(2.83, 4.72)	5.22***	(4.66, 5.77)
**Social participation**						
Socially active	(Ref)		(Ref)		(Ref)	
Socially inactive	0.16	(− 0.12, 0.44)	0.67***	(0.23, 1.12)	−0.12	(− 0.47, 0.24)
**Importance of religion**						
Not important	(Ref)		(Ref)		(Ref)	
Very important	1.03***	(0.84, 1.22)	0.77***	(0.46, 1.09)	1.19***	(0.95, 1.44)
**Living arrangement satisfaction**						
Not satisfied	(Ref)		(Ref)		(Ref)	
Neutral	2.93***	(2.54, 3.32)	3.58***	(2.94, 4.21)	2.53***	(2.04, 3.02)
Satisfied	5.91***	(5.55, 6.28)	6.21***	(5.60, 6.81)	5.72***	(5.26, 6.19)
**Faces discrimination in life**						
Yes	(Ref)		(Ref)		(Ref)	
No	1.63***	(1.42, 1.85)	1.43***	(1.05, 1.80)	1.72***	(1.46, 1.99)
**Received ill-treatment**						
Yes	(Ref)		(Ref)		(Ref)	
No	0.79***	(0.41, 1.18)	0.99***	(0.34, 1.64)	0.73***	(0.25, 1.21)
***Health characteristics***						
**Self-rated health**						
Poor	(Ref)		(Ref)		(Ref)	
Average	1.01***	(0.82, 1.20)	1.13***	(0.78, 1.48)	0.98***	(0.74, 1.21)
Good	0.98***	(0.70, 1.25)	0.99***	(0.51, 1.47)	1.01***	(0.67, 1.35)
**Chronic morbidity status**						
No condition	(Ref)		(Ref)		(Ref)	
Single condition	−0.055	(−0.23, 0.12)	−0.17	(− 0.47, 0.13)	0.010	(− 0.21, 0.23)
Multiple conditions	0.16	(−0.04, 0.36)	0.30	(−0.08, 0.68)	0.13	(−0.11, 0.37)
**Major depression**						
Depressed	(Ref)		(Ref)		(Ref)	
Not depressed	1.71***	(1.41, 2.01)	2.16***	(1.61, 2.71)	1.55***	(1.20, 1.91)
**Physical activity status**						
Physically active	(Ref)		(Ref)		(Ref)	
Physically inactive	0.17*	(−0.02, 0.35)	0.26*	(−0.04, 0.57)	0.10	(−0.13, 0.33)
**Difficulty in ADL**						
Faces difficulty	(Ref)		(Ref)		(Ref)	
No difficulty	0.10	(−0.10, 0.31)	0.17	(−0.19, 0.53)	0.060	(− 0.19, 0.31)
**Difficulty in IADL**						
Faces difficulty	(Ref)		(Ref)		(Ref)	
No difficulty	0.31***	(0.14, 0.48)	0.071	(−0.22, 0.36)	0.42***	(0.21, 0.63)
**Covered by health insurance**						
Yes	(Ref)		(Ref)		(Ref)	
No	0.60***	(0.41, 0.78)	0.63***	(0.33, 0.93)	0.59***	(0.36, 0.83)
***Household characteristics***						
**Household MPCE Quintile**						
Poorest	(Ref)		(Ref)		(Ref)	
Poorer	0.21*	(−0.02, 0.44)	0.34*	(−0.02, 0.70)	0.11	(−0.18, 0.41)
Middle	0.19	(−0.04, 0.42)	0.35*	(−0.02, 0.73)	0.088	(−0.21, 0.39)
Richer	0.10	(−0.13, 0.34)	0.11	(−0.29, 0.50)	0.061	(−0.24, 0.36)
Richest	0.084	(−0.16, 0.33)	−0.26	(− 0.69, 0.17)	0.15	(− 0.16, 0.46)
**Religion of household**						
Hinduism	(Ref)		(Ref)		(Ref)	
Islam	−0.26**	(− 0.50, − 0.03)	−0.42	(−1.01, 0.17)	− 0.23*	(− 0.50, 0.03)
Others	0.13	(− 0.11, 0.37)	− 0.23	(− 0.58, 0.12)	0.43**	(0.07, 0.79)
**Place of residence**						
Rural	(Ref)		(Ref)		(Ref)	
Urban	0.10	(−0.07, 0.28)	0.30*	(−0.03, 0.62)	0.036	(−0.17, 0.24)
**Country region**						
Southern	(Ref)		(Ref)		(Ref)	
Northern	0.64***	(0.42, 0.85)	0.91***	(0.48, 1.33)	0.50***	(0.24, 0.75)
Central	1.39***	(1.06, 1.72)	1.24***	(0.62, 1.85)	1.43***	(1.03, 1.83)
Western	3.16***	(2.91, 3.42)	3.61***	(3.12, 4.11)	2.93***	(2.62, 3.24)
Eastern	−0.038	(− 0.27, 0.20)	− 0.16	(− 0.62, 0.31)	0.0025	(− 0.28, 0.29)
North-eastern	0.65***	(0.36, 0.94)	0.98***	(0.50, 1.45)	0.53**	(0.11, 0.96)
**Adjusted R-squared**	**0.204**		**0.203**		**0.203**	
**Analytical sample size**	**30,370**		**9935**		**20,435**	

Note – (a) *CI* Confidence Interval, *(Ref)* Reference category; (b) Statistical significance denoted by asterisks where * *p*-value< 0.1, ** *p*-value< 0.05 and *** *p*-value< 0.01; (c) *ADL* Activities of daily living, *IADL* Instrumental activities of daily living, *MPCE* Monthly per capita consumption expenditure

Table 3 depicts the overall Blinder-Oaxaca decomposition of the caste differential in life satisfaction among older adults in India 2017-18. It was found that the model explained 74.3% of the caste gap (between SC/ST and non-SC/ST) in life satisfaction among older adults in India. However, 25.7% of the caste gap remains unexplained by the variables taken into consideration. This might be possible due to some unobserved factors which were not controlled during the analysis.

**Table 3:** Overall Blinder-Oaxaca decomposition of the caste differential in life satisfaction among older adults in India 2017-18

Component	Caste differential in life satisfaction		
	Coefficient	95% CI	Percent
**Explained Difference (E)**	−0.700***	(− 0.800, − 0.600)	74.3
**Unexplained Difference (U)**	− 0.242***	(− 0.413, − 0.071)	25.7
**Total Difference (T)**	− 0.941***	(−1.113, − 0.770)	

Note – (a) *CI* Confidence Interval; (b) Statistical significance denoted by asterisks where * *p*-value< 0.1, ** *p-*value< 0.05 and *** *p*-value< 0.01

Table 4 reveals the detailed Blinder-Oaxaca decomposition of the caste differential in life satisfaction among older adults in India 2017-18. Firstly, the differences due to explained component (E) are interpreted. A negative contribution presents that the factor was minimizing or narrowing the gap between SC/ST and non-SC/ST group and vice-versa. Subjective social status (39.0%) was widening the gap for life satisfaction among older adults from SC/ST and non-SC/ST group. Similarly, level of education (15.2%) followed by satisfaction with living arrangement (13.2%) and place of residence (5.3%) contributed for widening the gap for life satisfaction among older adults from SC/ST and non-SC/ST group. Region of country (− 11.5%) followed by self-rated health (− 3.0%) and major depression (− 2.7%) contributed for narrowing down the gap for life satisfaction among older adults from SC/ST and non-SC/ST group.

**Table 4:** Detailed Blinder-Oaxaca decomposition of the caste differential in life satisfaction among older adults in India 2017-18

Characteristics	Caste decomposition in life satisfaction among older adults					
	Explained component (E)			Unexplained component (U)		
	Coefficient	95% CI	Percent	Coefficient	95% CI	Percent
***Socio-demographic characteristics***						
**Age group**	−0.008***	(− 0.014, − 0.003)	0.9	0.057	(− 0.317, 0.432)	−6.1
**Gender**	0.002	(−0.001, 0.005)	− 0.2	0.174*	(− 0.021, 0.368)	−18.4
**Current marital status**	−0.008***	(− 0.014, − 0.002)	0.9	0.035	(− 0.197, 0.267)	−3.7
Level of education	− 0.143***	(− 0.183, − 0.104)	15.2	0.301	(− 0.091, 0.694)	− 32.0
**Working status**	− 0.013	(− 0.033, 0.007)	1.4	−0.597***	(− 0.986, − 0.208)	63.4
**Subjective social status**	− 0.367***	(− 0.405, − 0.329)	39.0	−0.027	(− 0.447, 0.394)	2.8
**Social participation**	0.002	(−0.004, 0.009)	−0.2	0.066**	(0.009, 0.123)	− 7.0
**Importance of religion**	−0.042***	(−0.055, − 0.029)	4.5	−0.442***	(− 0.758, − 0.125)	46.9
**Living arrangement satisfaction**	− 0.125***	(− 0.166, − 0.084)	13.2	−0.122	(− 1.009, 0.765)	13.0
**Faces discrimination in life**	0.011*	(−0.002, 0.025)	−1.2	−0.281	(− 0.684, 0.122)	29.8
**Received ill-treatment**	−0.002	(−0.007, 0.003)	0.2	0.380	(−0.438, 1.198)	−40.4
***Health characteristics***						
**Self-rated health**	0.028***	(0.016, 0.040)	−3.0	−0.088	(−0.672, 0.495)	9.4
**Chronic morbidity status**	−0.021	(−0.047, 0.005)	2.2	−0.017	(− 0.386, 0.352)	1.8
**Major depression**	0.026***	(0.015, 0.036)	−2.7	0.714**	(0.028, 1.399)	−75.8
**Physical activity status**	−0.004	(− 0.012, 0.004)	0.4	0.058	(−0.206, 0.321)	−6.1
**Difficulty in ADL**	−0.004	(−0.010, 0.002)	0.4	0.121	(−0.226, 0.469)	−12.9
**Difficulty in IADL**	0.006**	(0.001, 0.012)	−0.7	−0.260**	(− 0.467, − 0.053)	27.6
**Covered by health insurance**	− 0.033***	(− 0.044, − 0.021)	3.5	0.069	(−0.222, 0.359)	−7.3
***Household characteristics***						
**Household MPCE Quintile**	−0.023*	(−0.047, 0.002)	2.4	−0.144	(− 0.479, 0.190)	15.3
**Religion of household**	−0.040*	(−0.080, 0.000)	4.2	−0.370**	(− 0.690, − 0.050)	39.3
**Place of residence**	− 0.050***	(− 0.078, − 0.022)	5.3	0.054	(−0.052, 0.161)	−5.8
**Country region**	0.108***	(0.077, 0.140)	−11.5	−0.101	(−0.411, 0.210)	10.7
**Constant**	–	–	–	0.178	(−1.407, 1.762)	−18.9

Note – (a) *CI* Confidence interval; (b) Statistical significance denoted by asterisks where * *p*-value< 0.1, ** p-value< 0.05 and *** *p*-value< 0.01; (c) *ADL* Activities of daily living, *IADL* Instrumental activities of daily living, *MPCE* Monthly per capita consumption expenditure

## Discussion

The present study explored the caste differential in life satisfaction among older population in India using the secondary data of the LASI, wave-1. There was a significant difference between SC/ST and non-SC/ST groups in terms of life satisfaction. Older adults belonging to non-SC/ST group showed a higher mean score of life satisfaction than SC/ST group. In addition, the regression model showed a significantly higher likelihood of life satisfaction among non-SC/ST individuals as compared to SC/ST people. In India, the caste system continues to have a significant influence on people’s lives. It determines their social status as well as their access to a variety of resources throughout life [37]. This indicates the existence of caste-based socioeconomic disparities in the Indian society. The upward trend in socioeconomic indicators across the hierarchy of caste demonstrates how higher social status brings advantages and opportunities in life [38]. As a result, people who belong to the upper castes are more satisfied than those from the lower castes, like the SC/ST group.

The findings of the study indicated a positive association between older age and higher life satisfaction, both in case of SC/ST and non-SC/ST groups. The result is in line with the findings of a study from China, which showed a higher level of life satisfaction among older individuals than their younger counterparts [39]. However, it is inconsistent with the study by Kim & Sok [40]. The higher degree of life satisfaction among the elderly people can be explained by the phenomenon known as paradox of ageing [41]. With the increase in age, individuals develop a tendency to disregard irrelevant negative stimuli, respond less negatively to unfavourable events, and recall more positive information than negative information as they become older [42]. They even use fewer interpersonal comparisons than younger people. All these factors contribute positively to achieving a higher level of satisfaction in life among older adults.

In case of SC/ST group, the female population had shown greater likelihood of life satisfaction than their male counterparts. This corroborates with the findings by Ng et al. [43] and Zhou et al. [44], but contradicts with other studies that reported higher level of life satisfaction among older men [45]. One of the possible explanations could be the advantage of females in adopting to older age complications than males [46], who are more impacted by instrumental aspects of life. Further, marital status and level of education were found to be major contributors to caste differences in life satisfaction and are positively related to life satisfaction in both SC/ST and non-SC/ST groups. The analysis revealed that older adults with a marital status of “currently married” had higher life satisfaction. This is consistent with other studies [47, 48]. Marriage is linked to an increase in self-esteem, and married people are more likely to benefit from a supportive relationship [49]. This could be the possible explanation for the association between marital status and life satisfaction. Furthermore, more educated individuals were significantly more likely to have a higher degree of life satisfaction than their less educated counterparts. Also, education significantly contributed to the caste differences in life satisfaction (15.2%). This is consistent with previous studies [4, 50]. Education has an impact on socioeconomic status and life opportunities, and hence, life satisfaction is the outcome of the accumulated benefits [51].

Considering the association between working status and life satisfaction, our analysis indicated that retired individuals were more likely to show higher life satisfaction as compared to older adults who were currently working. One probable explanation could be the financial stability of retired people due to their pensions which give them financial security and resources to mitigate the challenges of life in older ages. In accordance with the findings of previous studies [47, 52], our study confirmed that subjective social status has a strong influence on life satisfaction of older adults, both among SC/ST and non-SC/ST groups. In the hierarchical culture of India, it is quite expected to find a greater level of life satisfaction among older adults enjoying a higher social status. The greater contribution of subjective social status to the caste differences in life satisfaction (39%) indicates the higher levels of caste-based discrimination that deprives individuals from lower castes of the modes of social capital, which is vital part of mental wellbeing as they age [33, 53]. It appears that people who compare their social status with others and find themselves in a lower position, are more prone to feel pessimistic and despairing [54]. This can make them unhappy and lead to a lower level of life satisfaction. Similarly, lower subjective social status is related to older individuals’ cognitive abilities [55], which can affect their level of wellbeing.

Further, the results of this study showed a significant and positive effect of religiosity on life satisfaction in older ages. However, the association is stronger among the non-SC/ST group than the SC/ST group. Several studies have also documented a higher level of life satisfaction among religious people than non-religious individuals [56]. Thus, alternative social support mechanisms such as religious participation can be promoted in terms of enhancing mental wellbeing among older Indian adults [57]. Furthermore, the study has found that living arrangement satisfaction plays an important role in determining life satisfaction in older age. In addition, older individuals who had never experienced any kind of discrimination or ill treatment were more likely to have a higher degree of life satisfaction.

Our findings also reflect earlier studies indicating that health plays a significant role in people’s perceptions of how satisfied they are with their life [43, 45, 50]. The present study has found poor self-rated health as a risk factor for a lower level of life satisfaction among older adults in both SC/ST and non-SC/ST groups. The finding is in agreement with previous studies reporting a significant positive association between self-rated health and life satisfaction [47, 58, 59]. However, our analysis did not show any significant association between co-morbidity and life satisfaction among older adults. The greater influence of perceived or subjective health status over objective health measures is consistent with earlier research [43, 60]. Another important factor that affects the life satisfaction of the elderly people is depression. Kim et al. [45], Onishi et al. [61], and Puvill et al. [62], have also reported a similar result. Good health boosts one’s self-esteem, sense of belonging, and gives a purpose to life, resulting in a greater degree of life satisfaction. Moreover, the significant contribution of self-rated health, depression and health insurance coverage to caste differences in life satisfaction provide insights that can be used to inform targeted policies and programs in the country.

Previous studies have shown that there is a significant rural-urban differential in terms of life satisfaction [43, 63]. In accordance with this finding, the present study has also documented a higher level of life satisfaction among urban elderly people belonging to SC/ST group than their rural counterparts. However, no significant association between place of residence and life satisfaction was found among non-SC/ST group. The caste system is still prevalent in rural India and people get discriminated on the basis of their caste. However, urbanization undermines caste. This could be one of the probable explanations for the higher likelihood of increased life satisfaction among urban elderly people belonging to SC/ST group. In addition, greater access to healthcare services, modern facilities, and good infrastructure in urban areas also explain the significant contribution of residential status to the caste differences in life satisfaction in this study.

Although the current findings are limited by the cross-sectional design of the study which prohibits from any causal inferences, they are indicative of societal and decision-making changes that may be in order. They imply that the caste system which is a type of social stratification in the Indian society; is a hurdle for social mobility and socioeconomic changes along with better health for individuals belonging to lower castes (SC/ST). On the other hand, individuals from comparatively higher castes (non-SC/CT) might acquire more power and privilege and have better health outcomes. Caste is strongly correlated with socioeconomic and health inequalities and low caste groups encounter significantly more deprivation than higher castes across India [64]. In spite of many changes that have occurred in modern India, the existence of discrimination among people becomes the basis of socioeconomic and health-related inequality. Discrimination based on caste is also associated with different value for individuals, leading to their unequal position in the social hierarchy and poor mental health and lower wellbeing outcomes [35]. Caste also impacts individuals’ educational and job opportunities as well as healthcare access which increases their vulnerability to health risks [65]. Therefore, the current findings can help identify the sub-populations with lower wellbeing among SC/ST groups and frame policies that can address their vulnerabilities.

## Conclusion

The findings demonstrated a significant caste differential in life satisfaction among older adults. Older adults belonging to non-SC/ST groups were more likely to have a higher level of life satisfaction than those from the SC/ST group. Factors like subjective social status, educational level, living arrangement satisfaction, and place of residence explained the caste differential in life satisfaction among older adults. In addition, factors such as psychological health and perceived health status should be the area of concern and special focus for policy makers and researchers in terms of reducing social inequalities in wellbeing indicators among older population. Proper designing of the policies and welfare programmes for older people needs a better understanding of the relationship between socioeconomic characteristics and individuals’ perceptions regarding life satisfaction.

## Data Availability

The study uses secondary data which is available in the public repository of International Institute for Population Sciences, Mumbai, accessible through. https://www.iipsindia.ac.in/content/lasi-wave-i.

## Abbreviations

SC: Scheduled Caste
ST: Scheduled Tribe
OBC: Other backward class
CI: Confidence interval
ADL: Activities of daily living
IADL: Instrumental activities of daily living
MPCE: Monthly per capita consumption expenditure
